# Positive Selection of Squalene Synthase in Cucurbitaceae Plants

**DOI:** 10.1155/2019/5913491

**Published:** 2019-05-09

**Authors:** Jieying Qian, Yong Liu, Chengtong Ma, Naixia Chao, Qicong Chen, Yangmei Zhang, Yu Luo, Danzhao Cai, Yaosheng Wu

**Affiliations:** ^1^Key Laboratory of Biological Molecular Medicine Research of Guangxi Higher Education, Department of Biochemistry and Molecular Biology, Guangxi Medical University, Nanning, Guangxi 530021, China; ^2^School of Medicine, Institutes for Life Sciences and National Engineering Research Center for Tissue Restoration and Reconstruction, South China University of Technology, Guangzhou, Guangdong 510006, China; ^3^School of Pharmacy, Guangdong Medical University, Dongguan, Guangdong 523808, China

## Abstract

Triterpenoid saponins are secondary metabolites synthesized through isoprenoid pathways in plants. Cucurbitaceae represent an important plant family in which many species contain cucurbitacins as secondary metabolites synthesized through isoprenoid and triterpenoid pathways. Squalene synthase (*SQS*) is required for the biosynthesis of isoprenoids, but the forces driving the evolution of *SQS* remain undetermined. In this study, 10 *SQS* cDNA sequences cloned from 10 species of Cucurbitaceae and 49 sequences of *SQS* downloaded from GenBank and UniProt databases were analyzed in a phylogenetic framework to identify the evolutionary forces for functional divergence. Through phylogenetic construction and positive selection analysis, we found that *SQS* sequences are under positive selection. The sites of positive selection map to functional and transmembrane domains. 180L, 189S, 194S, 196S, 265I, 289P, 389P, 390T, 407S, 408A, 410R, and 414N were identified as sites of positive selection that are important during terpenoid synthesis and map to transmembrane domains. 196S and 407S are phosphorylated and influence *SQS* catalysis and triterpenoid accumulation. These results reveal that positive selection is an important evolutionary force for *SQS* in plants. This provides new information into the molecular evolution of *SQS* within the Cucurbitaceae family.

## 1. Introduction

Plants encounter an array of pathogens and pests to which they induce preformed defense mechanisms including the synthesis of phytoalexin, semiochemicals, and terpenoids [1–4]. Triterpenoid saponins are secondary metabolites synthesized through isoprenoid pathways which enable plant defenses [5, 6] and have various clinical benefits including antitumor, anti-inflammatory, antiviral, cholesterol-lowering, and immune activation properties [7–14]. Squalene synthase (*SQS*) is a critical enzyme in the triterpenoid biosynthetic pathway [15–17], which is responsible for condensing two molecules of farnesyl diphosphate into squalene.

To adapt to environmental stress and development, the accumulation of squalene is regulated at various steps of the biosynthetic pathway [18]. Previously, it was reported that the overexpression of *Panax ginseng* squalene synthase 1 (*PgSS1*) results in enhanced biosynthesis of both phytosterols and triterpene saponins in *Panax ginseng* [19], which determines the content of saponin and other downstream products. Enhancing the activity of *PgSS1* increases the levels of phytosterols including ginsenoside, demonstrating that *PgSS1* is a key regulatory enzyme not only for phytosterol synthesis but also for triterpene biosynthesis. When *SQS* is transformed into the callus to produce transgenic plants, *SQS* activity is up to threefold higher than that of wild-type plants. This suggests that the accumulation of phytosterols and triterpenoids can be enhanced by metabolic engineering [20]. These results reveal that *SQS* is critical to the triterpenoid biosynthetic pathway and that *SQS* overexpression increases the biosynthesis of triterpenoids. In the Cucurbitaceae family, cucurbitane-type tetracyclic triterpenoids possess a variety of notable pharmacological activities [21]. To understand the mechanism(s) of cucurbitane biosynthesis, insight into the *SQS*-mediated triterpenoid biosynthetic pathway in Cucurbitaceae is required. To gain new understanding into the role of *SQS* during triterpenoid biosynthesis, we isolated and characterized 10 *SQS* cDNA clones from Cucurbitaceae plants (*Gynostemma pentaphyllum*, *Momordica charantia*, *Momordica cochinchinensis*, *Cucumis sativus*, *Benincasa hispida var. chieh-qua*, *Luffa acutangula*, *Cucurbita moschata*, *Trichosanthes rubriflos*, *Trichosanthes truncata*, and *Trichosanthes kirilowii*).

The regulation of gene expression within the triterpenoid synthetic pathway has gained a significant interest [22, 23]. Two or more copies of *SQS* in several plants have been identified, including *Trichosanthes truncata* C.B. Clarke, *Cucumis sativus* Linn, *Panax ginseng* [19], and *Arabidopsis thaliana* [24]. *SQS1* of *Panax ginseng* (*PgSS1*) overexpression resulted in enhanced biosynthesis of both phytosterols and triterpenoid saponins in *Panax ginseng* [16]. The ectopic expression of *PgSS1*, *PgSS2*, and *PgSS3* in the yeast erg9 mutant strain 2C1 lacking *SQS* activity restored ergosterol prototrophy [19].

Some results demonstrated that *SQS* is targeted to the ER membrane, and moreover, this location is exclusively dependent on the presence of the predicted C-terminal transmembrane domain [25]. The significance of *SQS* biological diversity suggests that it is subjected to positive Darwinian selection. Many research groups successfully rely on whole-gene random mutagenesis and recombination approaches for the directed evolution of enzymes, which have improved the properties of enzyme [26]. In the isoprenoid pathway for triterpenoid saponin synthesis, farnesyl pyrophosphate synthase (*FPS*) and *SQS* are the rate-limiting enzymes, which are considered to play an important regulatory role in the pathway. And our previous research found that FPS proteins in plants are under positive selection [27]. We want to investigate the force to evolution for *SQS* enzyme and explore the relationships between positively selected sites and some essential catalytic sites.

In this study, the squalene synthase of *Gynostemma pentaphyllum* (*GpSS*) served as a reference sequence for comparison with other plants. We identified two conserved sequences 77-82 (DTVEDD) and 213-217 (DYLED) important for the catalytic activity of isopentenyl pyrophosphate synthase (Trans-IPPS) and its downstream products. We assess how the evolution of *SQS* regulates its function and if the DTVEDD and DYLED motifs are positively selected. To investigate this, we cloned *SQS* sequences of 10 species in the Cucurbitaceae family and analyzed nucleotide and amino acid divergence in 59 plant species. We reveal important evolutionary functional sites of *SQS* through positive selection analysis. The likelihood method with site models, branch models, and branch-site models was used to calculate active amino acid sites and to investigate potential patterns of positive selection of the *SQS* gene.

## 2. Materials and Methods

### 2.1. Plant Material and Treatment

Plants of the Cucurbitaceae family were cultivated in a natural environment. Leaves of all Cucurbitaceae plants were collected in the summers of 2014 and 2015 from the Guangxi University of Chinese Medicine (*Momordica cochinchinensis*, *Trichosanthes rubriflos*, *Trichosanthes truncata*, and *Trichosanthes kirilowii*) and from the Guangxi Medical University (*Gynostemma pentaphyllum*, *Momordica charantia*, *Benincasa hispida var. chieh-qua*, *Luffa acutangula*, and *Cucurbita moschata*) in Guangxi Province, China. Fresh leaves were sampled, immediately frozen in liquid nitrogen, and stored at -80°C for RNA isolation. All the samples were authenticated by Prof. Ruisong Huang and Prof. Yaosheng Wu. The kits used in the experiments were as follows: Takara RNAiso Plus™ (Code No.: 9109), Takara PrimeScript™ II 1st Strand cDNA Synthesis Kit (Code No.: 6210A), Takara LA Taq DNA polymerase (Code No.: RR02MA), Takara 3′-Full RACE Core Set with PrimeScript™ RTase (Code No.: 6106), Takara MiniBEST Agarose Gel DNA Extraction Kit Ver.4.0 (Code No.: 9762), Takara MiniBEST DNA Fragment Purification Kit Ver.4.0 (Code No.: 9761), Takara Premix Taq™ Version 2.0 plus dye (Code No.: RR901A), and TransGen pEASY-T1 cloning kit (Code No.: CT101-02). Primers were synthesized by the IGE Biotech Company (Guangzhou City, China).

### 2.2. RNA Extraction

Total RNA was extracted from the plant leaves of Cucurbitaceae using TRIzol. RNA quantity was determined on a NanoDrop 2000 Spectrophotometer (Thermo Scientific, San Jose, California, USA). RNA integrity was analyzed by electrophoresis on 1.5% agarose gels and the purity and concentration assessed. RNA (1 *μ*g) from each sample was reverse transcribed in a final volume of 20 *μ*L using the Takara 3′-Full RACE Core Set with the PrimeScript™ RTase synthesis kit.

### 2.3. cDNA Cloning

Takara RACE Kits (3′-Full RACE Core Set with PrimeScript™ RTase) were used to clone full-length cDNA which was amplified by PCR and cloned into the pEASY-T1 cloning vector. The vector was transformed into Trans-T1 chemically competent cells and cultured in Lysogeny Broth (LB) medium at 37°C. Positive plasmids were extracted and identified by double restriction digest (QuickCut™ BamH I and QuickCut™ Sac I). Positive clones were sent to Sangon Biotech Company (Shanghai City, China) for sequencing. According to the 3′-RACE cDNA sequence, 5′-RACE primers were designed and PCR was performed (SMART 5′-RACE and 3′-RACE). Using Vector NTI 6.0 software, the sequences of 3′-RACE and 5′-RACE amplification products were spliced, and full-length cDNA sequence of 10 *SQS* genes from Cucurbitaceae plants was obtained.

### 2.4. Sequence Data

The sequence datasets consisted of *GpSS* (FJ906799.1) of *Gynostemma pentaphyllum*, *PnSS* (DQ186630) of *Panax notoginseng*, and nine *SQS* sequences from Cucurbitaceae plants which were cloned using RACE technology, in addition to 48 *SQS* cDNA and amino acid sequences downloaded from GenBank (http://www.ncbi.nlm.nih.gov/) and UniProt databases (http://www.uniprot.org/). Sequences were BLAST searched, and only full-length coding sequences were assessed in final analysis. In addition, each corresponding protein matched its target CDs. The final datasets consisted of 59 *SQS* sequences of terrestrial plants, including 2x Pteridophyte, 2x Gymnosperms, 7x Monocotyledons, and 46x Dicotyledons. *Botryococcus braunii* and *Selaginella moellendorffii* served as outgroups.

### 2.5. Sequence Alignment

All protein sequences were aligned in MUSCLE [28] using the default parameters (http://www.ebi.ac.uk/Tools/msa/muscle/), and PAL2NAL [29] (http://www.bork.embl.de/pal2nal/) was used to align amino acids and rearranged CDs. To convert nucleotide sequences to the nexus format, we ran MEGA4.0 [30] which allowed phylogenetic analysis in the MrBayes version 3.1.2 [31, 32]. The parameters in the nexus file with PAUP∗ version 4.0 [33] were modified with the Model test version 3.7 [34] prior to MrBayes calculation. However, recombination events and substitution saturation can cause failure to reconstruct the phylogenetic tree and influence the detection of positive selection. Therefore, prior to performing reconstruction of the tree and positive selection, we must assess the recombination signals and substitution saturation between sequences involved in the alignment of *SQS* gene sequences. The Genetic Algorithm for Recombination Detection (GARD) approach [35] was applied to screen multiple sequence alignments for evidence of phylogenetic incongruence and to identify the number and location of breakpoints and sequences involved in putative recombination events [36]. We applied DAMBE as a new index to measure substitution saturation in a set of aligned nucleotide sequences [37]. With all parameters modified, MrBayes was performed to reconstruct the phylogenetic tree [32]. According to the tree and aligned DNA sequences (CDs) in the DAMBE, PAML was performed for selected pressure analysis.

### 2.6. Positively Selected Sites and Putative Biological Significance

**Figure 1:**
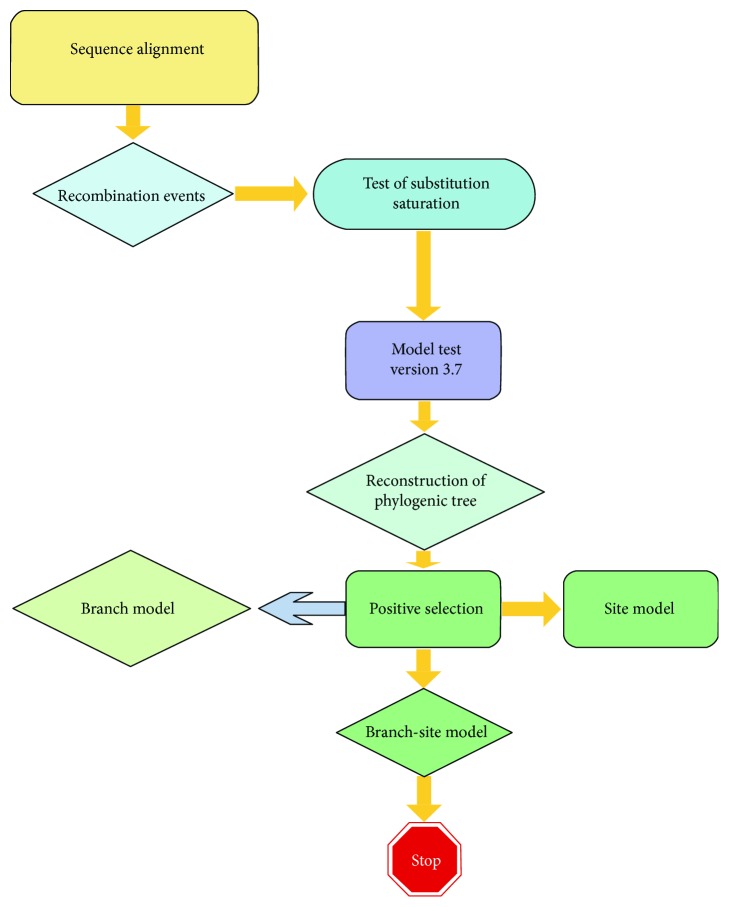
Technical routes of positive selection analysis in the site model, branch model, and branch-site model for *SQS* sequences in plants.

Based on the reconstruction of the phylogenetic tree of *SQS* with MrBayes, we performed positive selection analysis. Selection pressure was assessed based on the phylogeny of *SQS* gene sequences by comparing nonsynonymous (dN)/synonymous (dS) substitution ratios (*ω* = dN/dS) with *ω* = 1, *ω* < 1, and *ω* > 1, which indicated neutral evolution, purifying selection, or positive selection, respectively, prompting the formation of distinct subclasses and new functions in different species [38]. To explore selection pressure between *SQS* gene sequences, we performed strict statistical analysis using the CodeML program in the PAML package version 4 [39], which mainly runs site models, branch models, and branch-site models based on dN/dS(*ω*) [40]. The likelihood ratio test (LRT) [41] was used to compare fit models for data from two nested models to assess the statistical significance of each pair of nested models. Concrete methods have been previously described [27]. The analysis route of positive selection is shown in Figure 1. We used LRT through the comparison of M0 (one ratio) with M3 (discrete) (Genetic Algorithm for Recombination Detection = 3) to test for variable selection pressure amongst sites and two LRTs to test for sites evolving by positive selection, comparing M1a (nearly neutral) against M2a (positive selection) and M7 (beta) (*κ* = 10) against M8 (beta and *ω*) (*κ* = 10). A significantly higher likelihood of the alterative model compared to the null model suggested positive selection. Generally, all positive selection sites were calculated in the M8, which provided useful information for branch-specific and branch site analysis. The site models did not detect positive selection and influenced only selected sites along a small number of lineages following a duplication event, and so, the branch model and two-ratio test were implemented to select statistically significant “foreground branches” under positive selection. All other branches in the tree were classed as “background” branches. Based on the evolutionary branches screened out using the two-ratio test, branch-site models were applied to further estimate different dN/dS values amongst significant branches detected by the branch model and amongst sites [42]. Two models were computed: a null model (H0), in which the foreground branch differed according to the proportion of sites under neutral selection compared to background (relaxed purifying selection), and an alternative model (H1), in which the foreground branch has a proportion of sites under positive selection. The null and alternative hypotheses were as follows: null hypothesis (branch-site model, with *ω*2 = 1 fixed): model l = 2, NSsites = 2, fix_*ω* = 1, and *ω* = 1 and alternative hypothesis (branch-site model, with *ω*2 estimated): model = 2, NSsites = 2, fix_*ω* = 0, and *ω* = 1 (or any value > 1). Finally, the Bayes empirical Bayes (BEB) approach [41] was used to calculate posterior probabilities that a site originates from the site class with *ω* > 1, which was used to identify sites under positive selection or relaxed from purifying selection in the foreground group with significant LRTs.

### 2.7. Characteristics of Protein *SQS* Sequence and Structural Analysis

We used GeneDoc tools (http://www.nrbsc.org/gfx/genedoc) for visualizing, editing, and analyzing multiple sequence alignments of *SQS* proteins. We used NetPhos 2.0 Servers (http://www.cbs.dtu.dk/services/NetPhos/) to predict phosphorylation sites. PredictProtein [43, 44] and TMHMM2.0 [45] were used to predict transmembrane domains and posttranslational modifications. An estimate of the prediction accuracy was provided based on the confidence score of the modeling. Furthermore, the functional area and relevant positive selection sites identified in the evolutionary analysis were built in three-dimensional graphic models and presented in the highlight areas. Finally, the Discovery Studio Visualizer (DSV) [46] designed to validate any predictions of protein-ligand interactions was used to reveal sites under positive selection in the *SQS* 3D structure. Meanwhile, we combined positively selected sites with proteomic analysis to investigate their potential relationship.

## 3. Results

### 3.1. Full-Length cDNA Sequences Cloned from Cucurbitaceae Plants

Full-length cDNA sequences of *SQS* in Cucurbitaceae were obtained from *Gynostemma pentaphyllum*, *Momordica charantia*, *Momordica cochinchinensis*, *Cucumis sativus*, *Benincasa hispida var. chieh-qua*, *Luffa acutangula*, *Cucurbita moschata*, *Trichosanthes rubriflos*, *Trichosanthes truncata*, and *Trichosanthes kirilowii*. ORF finder showed that all cloned sequences had ORFs of 1254 bp encoding a protein 417 amino acids in length. For the prediction of the physical and chemical properties of *SQS* gene sequences of Cucurbitaceae, we analyzed the theoretical pI and molecular weight (kD) of these sequences (Table 1). We found that the pIs ranged from 7.50 to 8.50 and the molecular weights were ~47 kD. All Cucurbitaceae *SQS* sequences were used for phylogenetic analysis.

### 3.2. Origin of the *SQS* Sequences during Plant Evolution

**Figure 2:**
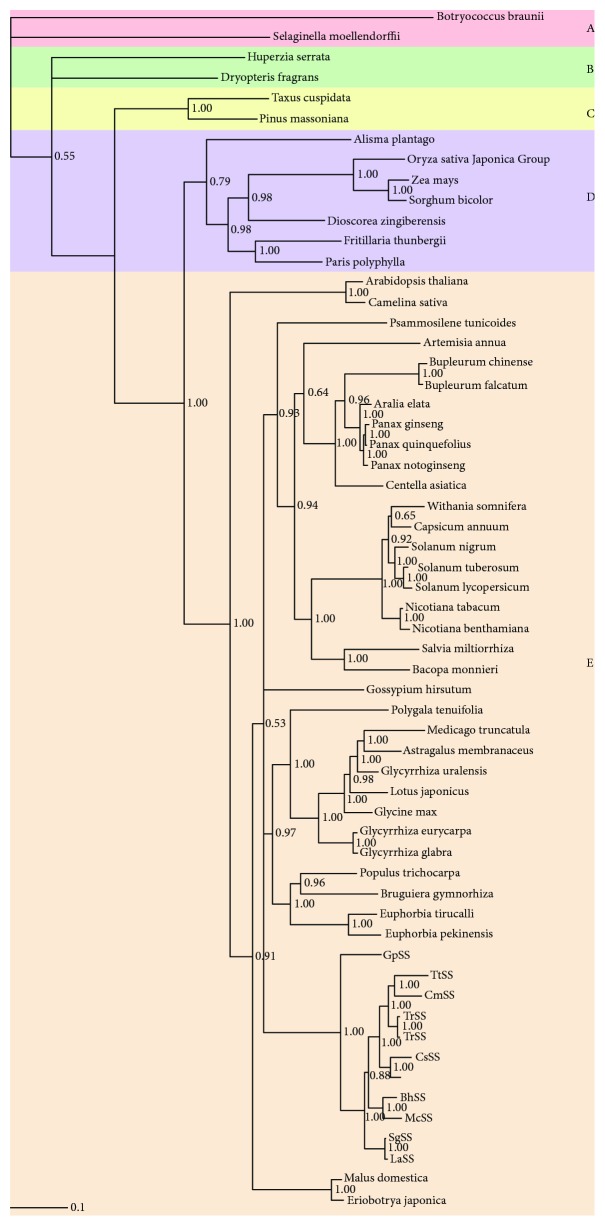
Phylogenic tree of terrestrial plant *SQS* was inferred by Bayesian analyses and divided into five clades. *Botryococcus braunii* and *Selaginella moellendorffii* were used as outgroup A. Pteridophyte, Gymnosperms, Monocotyledons, and Dicotyledons were labeled as groups B, C, D, and E, respectively. Posterior probabilities are labeled above branches. Numbers indicate the Bayesian probabilities for each phylogenetic clade. Abbreviations of *GpSS*, *TtSS*, *CmSS*, *TrSS*, *TkSS*, *CsSS*, *BhSS*, *MchSS*, *McSS*, *SgSS*, and *LaSS* represent the squalene synthase of *Gynostemma pentaphyllum*, *Trichosanthes truncata*, *Cucurbita moschata*, *Trichosanthes rubriflos*, *Trichosanthes kirilowii*, *Cucumis sativus*, *Benincasa hispida var. chieh-qua*, *Momordica charantia*, *Momordica cochinchinensis*, *Siraitia grosvenorii*, and *Luffa acutangula*, respectively.

The phylogenetic tree was reconstructed to investigate selection pressure amongst the 59 plant sequences. Through GARD detection, no recombination events occurred within the *SQS* sequences. Substitution saturation showed that the Iss < Iss.c was significantly different, indicating a little saturation between the *SQS* sequences. It was therefore inferred that the phylogenetic tree reconstruction and positive selection of *SQS* are not influenced by recombination and substitution saturation. To investigate the phylogenetic relationship amongst *SQS* sequences, a phylogenetic tree based on codon alignment was produced using the Bayesian method (Figure 2). Bayesian posterior probability (pp) methods were used to evaluate clades. Phylogenetic analysis showed that *SQS* sequences consisted of several distinct branch clusters that accompanied species divergence [47], which could be grouped into five lineages (Outgroup, Pteridophyte, Gymnosperms, Monocotyledons, and Dicotyledons) with *Botryococcus braunii* and *Selaginella moellendorffii* emerging as outgroups (group A in Figure 2). The relationships displayed in the phylogenetic tree were identical to the taxonomic classifications. Thus, the phylogenetic tree of the *SQS* sequences of terrestrial plants reflects the genetic relationship amongst different species. *SQS* sequences emerged in branch points of the terpenoid synthesis pathway, which is responsible for directing carbon flow from the isoprenoid pathway [48]. The reaction products catalyzed by *SQS* may therefore flow into the triterpenoid metabolic pathway. According to the phylogenic tree, three obvious branches with high pp supportive values (1.00) were evident and included Gymnosperms, Monocotyledons, and Dicotyledons. In the Dicotyledoneae (group E in Figure 2), the first to emerge were *Arabidopsis thaliana* and *Camelina sativa*, belonging to Brassicaceae. *Malus domestica* and *Eriobotrya japonica* are the typical plants of Rosaceae in the phylogenic tree. The *SQS* sequences of the four species in Brassicaceae and Rosaceae suggested sharing of a common ancestral *SQS* gene that originated from monocots. Due to duplication and gene diversification of the *SQS* sequences, each branch constantly evolved in the Dicotyledoneae, based on the tree lineages. The evolution of *SQS* sequences in the plants accompanied divergence of the metabolic pathways. For example, the clades amongst *Panax ginseng*, *Panax notoginseng*, and *Panax quinquefolius* were clustered, suggesting that they share similar functions to that of Araliaceae *SQS*, which encodes enzymes participating in the biosynthesis of dammarane-type ginsenoside. The phylogenic tree of cloned *SQS* sequences of *Gynostemma pentaphyllum*, *Siraitia grosvenorii*, and other *SQS* sequences of Cucurbitaceae plants occurred at the same branch. Tetracyclic triterpenoids are also the main secondary metabolic substances in Cucurbitaceae. The saponins found in *Gynostemma pentaphyllum* mainly belong to the dammarane-type tetracyclic triterpenoids. The dammarane-type tetracyclic triterpenoids present in both Araliaceae and Cucurbitaceae may be closely related to triterpenoid synthesis and *SQS* enzyme activity.

### 3.3. Positively Selected Sites amongst *SQS* Subfamilies and Putative Biological Significance

We next analyzed whether positive selection occurred during lineage-specific evolution. We calculated the nonsynonymous/synonymous substitution ratio (dN/dS = *ω*) in *SQS* sequences using the PAML package version 4.4. Following the removal of gaps, *Gynostemma pentaphyllum* squalene synthase (*GpSS*) was used as the reference sequence amongst the 59 species, and all protein sequences were analyzed using the CodeML program. For site model detection, M0 vs. M3 and M1a vs. M2a showed more than a 95% occurrence of synonymous changes relative to nonsynonymous changes (dN/dS < 1), indicating that they were under a strong purification selection. However, the pairs of models of M7 vs. M8, the LRT (2ΔlnL = 11187.62, df = 2, *p* ≤ 0.01), showed a *ω* (dN/dS) greater than 1 in M8 (dN/dS > 1), indicating an excess of nonsynonymous changes. This result showed that positive selection had driven *SQS* evolution, which was distinguished from M0 vs. M3 and M1a vs. M2a. We detected 45 positively selected sites (Table 2) with a pp ≥ 99%. When considering positive selection only occurring during specific evolution stages or in specific branches, we used a branch-specific model to detect positive selection. The free ratio model was significantly higher than the one-ratio model (2ΔlnL = 256.64, *p* ≤ 0.01, df = 135), indicating a heterogeneous selection in 6 branches (*ω* > 1) (Table 3). Amongst these 6 branches (Ta, Tb, Tc, Td, Te, and Tf), a two-ratio model test was applied to screen out the evolutionary branches. Four branches (Ta, Tb, Te, and Tf) had a *ω* ≥ 1, indicating a strong positive selection in branches Ta, Tb, Te, and Tf (Table 4). The branch-site model was used to screen for amino acid sites that underwent positive selection in branches Ta, Tb, Te, and Tf. According to the LRT of the branch-site model, comparisons of BSa1 vs. BSa0-fix (2ΔlnL = 4.18, *p* < 0.05, df = 1), BSb1 vs. BSb0-fix (2ΔlnL = 7.02, *p* < 0.01, df = 1), BSe1 vs. BSe0-fix (2ΔlnL = 6.64, *p* < 0.01, df = 1), and BSf1 vs. BSf0-fix (2ΔlnL = 3.3, *p* > 0.05, df = 1) significantly differed. Interestingly, 13 amino acid sites in the branches displayed a least posterior probability of >0.95 by BEB (13D, 278D, and 196S with the pp > 0.95 in branch Ta, *p* < 0.01). At the same time, we detected positively selected sites 52A, 157K, 171Y, 180L, 203L, 226M, 289P, and 333G with the pp > 0.981 (*p* < 0.01) in the branch Tb and amino acid sites at position 37W and 308R with pp > 0.96 (*p* < 0.01). Interestingly, 196S was detected both in the site model and branch-site model, which was located in the phosphorylation site. All of these sites were considered to undergo positive selection. In our previous studies, *MchSS* and *TrSS* were listed as the first reference sequences for the calculation of positive selection amongst *SQS* sequences. Surprisingly, the distributions of positively selected sites of *SQS* from Cucurbitaceae were consistent. Moreover, the important functional site 196S was detected in different species of Cucurbitaceae. This indicated a strong positive selection amongst *SQS* sequences in Cucurbitaceae (Table 5). Further analysis showed that (1) the *SQS* gene underwent positive selection during the plant evolutionary process, particularly in Cucurbitaceae, and that (2) some representative positively selected sites emerged in the catalysis region and significant functional sites. These features suggested that positively selected sites located in the functional domains of *SQS* are important components of the *SQS* structure.

### 3.4. Structural Characteristics of *SQS* in Plants

**Figure 3:**
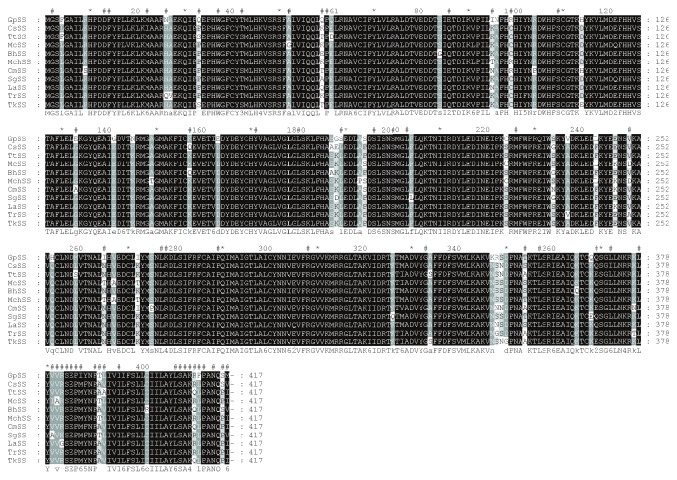
Multialignment of partial terrestrial plant *SQS* amino acid sequences. Sites of positive selection of *SQS* above 11 common medicinal Cucurbitaceae plants are shown through the Gendoc software, and *GpSS* was used as the reference sequence. Conserved sites are shaded, and the meaning of each symbol is given in the specific symbol. ^#^Positively selected site.

**Figure 4:**
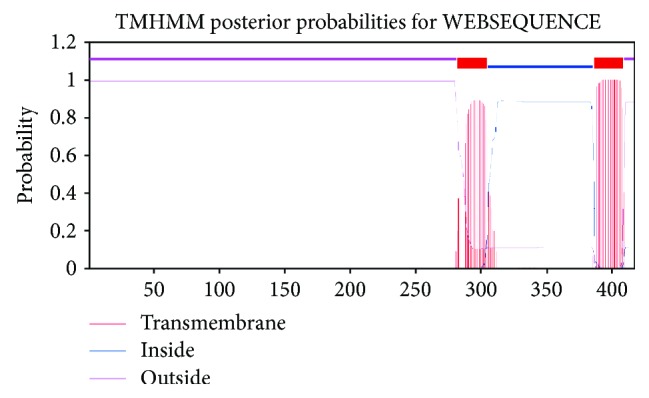
TMPRED and TMHMM predictions for transmembrane proteins. Two transmembrane regions (285-301 and 389-408) displayed a probability ≥ 0.8 and a score that exceeded 500, strongly indicating the presence of transmembrane domains.

**Figure 5:**
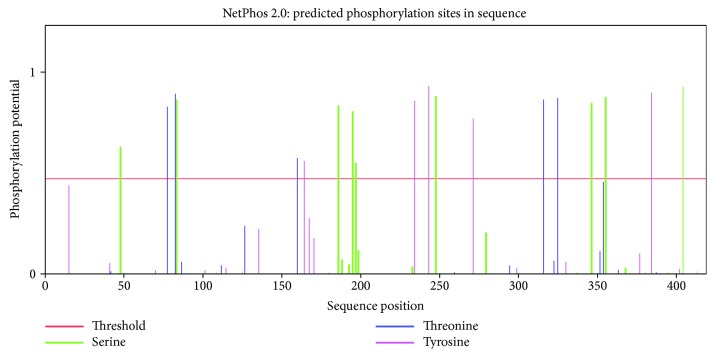
Prediction of phosphorylation sites in *GpSS*. These include 48S, 84S, 187S, 196S∗, 198S, 249S, 349S, 358S, and 407S∗; 78T, 83T, 161T, 318T, and 327T; and 165Y, 236Y, 246Y, 273Y, and 387Y.

In addition to the phylogenetic tree and positive selection analysis of *SQS* sequences, we conducted detailed structural studies from the two-dimensional model containing the sequence alignment of the *SQS* protein sequences in several medicinal herbs, including *GpSS*. *GpSS* was considered the reference sequence in this analysis. The *SQS* sequences shared a high level of sequence similarity within the coding region. We mapped positively selected sites through assessing sequence alignments of the *SQS* gene sequences amongst *GpSS*, *SgSS*, *MchSS*, *McSS*, *CsSS*, *BhSS*, *LaSS*, *CmSS*, *TrSS*, *TtSS*, and *TkSS* (Figure 3). As shown in the two-dimensional structure produced by GeneDoc, the positively selected 189S was located in the casein kinase II phosphorylation site on *GpSS* but at position 189L in the other 10 Cucurbitaceae plants. The positively selected sites 408A and 410R overlapped with the protein kinase C phosphorylation sites. This was 410R on *GpSS* but 410Q on *CmSS*, *BhSS*, *TtSS*, and *TkSS*. Moreover, positively selected 196S was a serine phosphorylation site, which was consistent in *SQS* in Cucurbitaceae plants. Based on these positive signal and functional sites, we inferred that the phosphorylation of these sites plays an important role in *SQS* activity, potentially influencing the further metabolic flux of squalenes. The positively selected 414N was a glycosylation site in the transmembrane region, which may have been related to *SQS* function. All these sites require further verification by site-directed mutagenesis in future studies. At the same time, TMHMM (http://www.cbs.dtu.dk/services/TMHMM-2.0) and TMPRED (http://www.ch.embnet.org/software/TMPRED_form.html) were used to predict the transmembrane domains of *GpSS*, and motif elicitation (MEME) analysis (http://meme-suite.org/) was used to obtain characterized motifs [49]. It is known that *SQS* is membrane-associated [15]. According to TMHMM and TMPRED predictions, two transmembrane helices are present in *GpSS*, located at position 285-301 (inside-outside, 1336 score) and position 389-408 (outside-inside, 2549 score) (Figure 4). These transmembrane domains indicated that *GpSS* is an integral membrane protein. The second transmembrane region (389-408) may have important biological roles in anchoring to *GpSS* to the ER membrane [24]. When MEME was employed, we identified two significantly conserved motifs (168-183 YCHYVAGLVGLGLSKL and 201-229 MGLFLQKTNIIRDYLEDINEIPKCRMFWP). These were located in the functional domains in *GpSS*. Through subcellular localization predictions, it was revealed that *GpSS* has no mitochondrial targeting signal or chloroplast transit peptides. Conserved domain analysis (http://www.ncbi.nlm.nih.gov/Structure/cdd/wrpsb.cgi) was performed to identify conserved domains [50]. This showed that *GpSS* belongs to the Trans_IPPS_HH superfamily (38-320 interval, *p* = 1.38*e* − 84) with substrate-binding pockets, Mg^2+^-binding sites, active site lid residues, catalytic residues, and an aspartate-rich region. NetPhos 2.0 Server was used to predict *SQS* phosphorylation sites which may regulate *SQS* activity (Figure 5). We identified 9 serine residues (48S, 84S, 187S, 196S∗, 198S, 249S, 349S, 358S, and 407S∗), 5 Thr (78T, 83T, 161T, 318T, and 327T), and 5 tyrosine residues (165Y, 236Y, 246Y, 273Y, and 387Y). Of these sites, 196S and 407S were positively selected.

### 3.5. Distribution of Positive Selection Sites on the *SQS* 3D Structure

**Figure 6:**
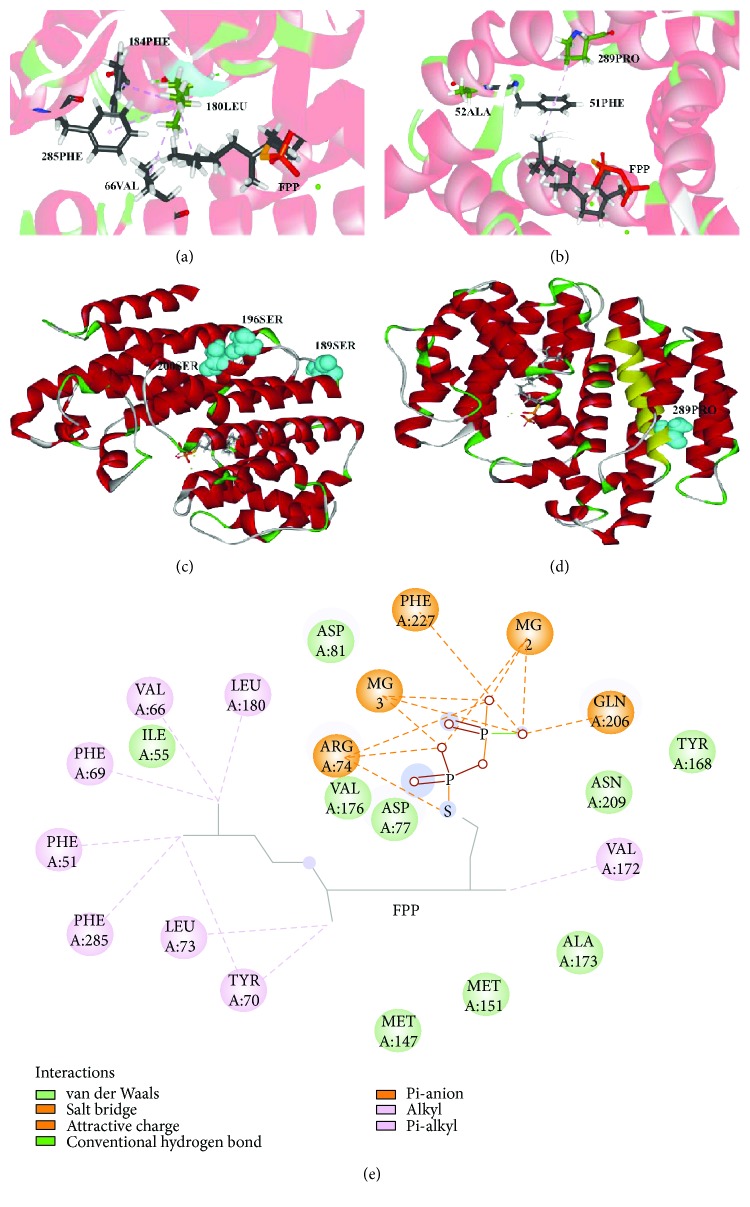
Sites of positive selection (52A, 180L, 189S, 196S, 200S, and 289P) on the *GpSS* model using DSV. (a) 180Leu was connected with FPP, 66Val, 184Phe, and 285Phe by nonbonded interactions. (b) 52Ala and 289Pro were connected with FPP and 51Phe. (c, d) Distribution of 189Ser, 196Ser, and 200Ser and 289Pro on the *GpSS* 3D structure. Yellow areas indicate transmembrane domains. (e) Relationship between *GpSS* receptors and ligands (FPP). Dashed lines indicate nonbonded interactions.

Due to evidence of the positive selection sites within the *SQS* sequences, the crystal structure of the human *SQS* (3vj8.1A) was used as a template to predict the 3D structure of *GpSS* using homology structural modeling on a Swiss model server [51]. Human *SQS* is a 47 kD membrane-associated enzyme that catalyzes the dimerization of two molecules of FPP in a two-step reaction to form squalene [52]. We used the structure of *GpSS* as a reference to analyze the relationship between positive selection sites and functional sites. We mapped important sites of positive selection from the branch-site model and site model onto the 3D structure using DSV. Whilst sites of positive selection mainly exist in the transmembrane domains indicating that *GpSS* undergoes strong constraints within its functional domains, important sites of positive selection (52A, 180L, 189S, 196S, 200S, and 289P) mapped to other areas (Figure 6). A residue with the *SQS* catalytic center (180L) was connected with the substrate FPP, in addition to 66V, 184F, and 285F by noncovalent bonds (Figures 6(a) and 6(e)). In Figure 6(b), a positively selected site 52A located near the catalytic sites 51F and 289P impacts on the condensation of two side chains of FPP by noncovalent interaction with 51F. Position 196S was identified as a phosphorylation site in both the site model and branch-site model, whilst 189S lies within a casein kinase II phosphorylation (Figure 6(c)) [52]. Moreover, 196S and 200S lie close to the first transmembrane domain, and the 289P lies within the first transmembrane domain (Figure 6(d)) [52].

## 4. Discussion

In this study, we obtained 10 cDNA sequences of *SQS* genes from 10 plants of the Cucurbitaceae family. To gain new insight into the role of *SQS* during the triterpenoid biosynthesis in higher plants, we performed phylogenetic analysis and evaluated sites of positive selection. The analysis revealed that *SQS* sequences in Cucurbitaceae plants are highly conserved and cluster into subfamilies. *SQS* sequences from different species of plants often exhibit both characteristic sequences. For example, three *SQS* genes are differentially expressed in *Panax ginseng*, all of which are involved in the generation of squalene in *Panax ginseng*. The enhanced activity of *PgSS1* results in increased levels of phytosterols and ginsenosides [16]. *PgSS* contains three conserved domains ((a) 168~183, (b) 202~224, and (c) 280~298) essential for the two half reactions catalyzed by *SQS* [19]. The three domains are also present in *GpSS* and other *SQS* genes of Cucurbitaceae plants. The amino acid sequences within these *SQS* domains are identical, and variation exists only in the first two amino acids. This may explain why dammarane-type tetracyclic triterpenoids are present in the *Panax* genus and some Cucurbitaceae plants, particularly in *Gynostemma pentaphyllum*. Conserved protein domain analysis through PredictProtein (https://www.predictprotein.org/) revealed that *GpSS* and all other Cucurbitaceae *SQS* genes contain an isopentenyl diphosphate synthase- (Trans_IPPS-) conserved catalytic center that consists of a large central cavity formed by antiparallel *α*-helices with two aspartate-rich regions (DXXXD). The results indicate that typical *GpSS* from Cucurbitaceae plants possess two transmembrane domains at the C-terminus (285-301 AA and 385-408 AA), which is consistent with the properties of other eukaryote *SQS* proteins bound to the ER membrane [53]. The aspartate-rich motifs in Cucurbitaceae *SQS* were located at residues 77 to 82 (DTVEDD) and 213 to 217 (DYLED). These motifs underwent a strong negative selection pressure at the constraints which were highly conserved. The motifs were conserved during evolution to maintain the basic functions of *SQS* proteins. We found no sites of positive selection located within these highly conserved motifs. Thus, positive selection events primarily occur outside the active center of *SQS*, consistent with the negative selection of the active center.

As the number of *SQS* genes cloned in our laboratory and collected from the databases is growing, it is feasible to explore both evolutionary relationships and the genetics of the *SQS* family. In this study, 59 sequences were used for phylogenetic tree reconstruction using Bayesian methods. It was shown that triterpenoid saponins are widely distributed in dicotyledonous plants [3, 26, 54–56]. Cucurbitaceae resident tetracyclic triterpenoids are valuable active ingredients for self-defense and possess many clinical values when extracted. Thus, studying the molecular traits of the *SQS* triterpenoid pathway of Cucurbitaceae plants is important. Gene expression analysis revealed that *SQS* increases terpenoid generation in plants [57–59]. However, the mechanism(s) by which *SQS* regulates this pathway and its species-specific biological roles remain poorly understood. Using molecular adaptive evolution and positive selection principles to identify functional sites can provide valuable references to the artificial regulation of triterpenoid synthesis.

In this study, we combined molecular phylogenetic trees with putative biological significance and protein structure analysis to explore the evolution of terrestrial plant *SQS*. Positive selection results in the retention and spread of advantageous mutations throughout a population and has long been considered synonymous with protein functional shifts [60]. The previous study found that positively selected genes were likely to interact as opposed to interactions with genes under neutral evolution or purifying selection [61]. To investigate evidence for the positive selection of *SQS* in the Cucurbitaceae family, we analyzed the site model, branch model, and branch-site models. Analysis of each of the subfamilies under the site model (M0 vs. M3, M1a vs. M2a, and M7 vs. M8) detected several sites of positive selection for all the subfamilies, suggesting that selective pressure varies amongst lineages and amino acid sites in *SQS* genes. From positive selection analysis, the category of *ω* in the site model did not sufficiently fit the data to a level that describes the variability in selection pressure, whilst branch model analysis showed variable *ω* ratios amongst clades. The branch-site model was applied to evaluate sites amongst specific clades of the *SQS* phylogeny.

As shown in the space structural model of *GpSS* (Figure 6), 189S is located within a casein kinase II phosphorylation site, 408A and 410R are located in protein kinase C phosphorylation sites, 414N lies within a glycosylation site, and 389P, 390T, 391L, 394I, 406L, 407S, and 408A are in the second transmembrane domain (389-408) (Figure 4). Importantly, 196S and 407S are also phosphorylation sites (Figure 5). As detected in the branch-site model, 289P is located in the first transmembrane domain (285-301). 196S was detected in the site model and branch-site model and considered as a vital site of *SQS*-mediated triterpenoid synthesis. Given that phosphorylation/dephosphorylation regulates enzyme activity, this site is predicted to mediate *GpSS* and impact on triterpenoid biosynthesis.

Although the active center of *SQS* from various species is highly conserved, distinct sites were also reported. *LjSS* (*Lotus japonicas* squalene synthase) possessed an unusual Asp (D280) near the active center. Site-directed mutagenesis and *in vitro* assessment of *LjSS* demonstrated that D280 can be substituted by Gln and Glu, suggesting that the local structure of plant *SQS* differs from mammalian *SQS* in which Gln at the same site cannot be replaced by Glu to retain activity [62]. Sequence alignment demonstrated that D280 is located in all known Cucurbitaceae *SQS* proteins near the active center. Two sites of positive selection include L178 and K179 for *GpSS* but are L178 and R179 for other known Cucurbitaceae *SQS* proteins.

Two transmembrane domains (285-301 and 389-408) are present on the Cucurbitaceae structure. Our analysis indicated that 289P, a site of positive selection, is located in the first transmembrane region. On the second transmembrane region, other sites of positive selection, namely, 389P, 390T, 391L, 394I, 406L, 407S, and 408A, are present. *SQS* should contain sequences responsible for its localization on the ER membrane [53]. The sites of positive selection on the second transmembrane region would enhance ER targeting and thus promote cellular *SQS* activity. The majority of the sites mapped to this region. Pathway evolution indicated that genes that regulate similar functional pathways maintain a consistent or associated evolutionary trend [63, 64].

It was reported that positive gene selection during species evolution drives diversification during plant secondary metabolism [65]. Many of the genes that undergo positive selection tend to interact [61]. In the previous studies, the positive selection was reported in plant farnesyl pyrophosphate synthase (FPS). We found that the FPS genes of terrestrial plants also undergo positive selective pressure, generating a functional diversity [66]. FPP, produced through the catalytic activity, is the *SQS* substrate. Both FPS and *SQS* are key enzymes in triterpenoid biosynthesis. Thus, why both *FPS* and *SQS* experience positive selection pressure is worthy of further exploration. Based on FPS and *SQS* structural analysis, both were found to contain two isopentenyl diphosphate synthase- (Trans_IPPS-) conserved catalytic sites, including *SQS* head-to-head (Trans_IPPS_HH) [52] and FPS head-to-tail (Trans_IPPS_HT) [67]. These associated structures may determine the flux of metabolites catalyzed by *SQS* and FPS, in addition to other enzymes associated with triterpenoid biosynthesis. The formation of large complexes may be regulated by the metabolic requirement, in which the previous enzyme acts as a substrate for the next enzyme. This may be more beneficial to the formation of secondary metabolites and structural diversity. Thus, we provide new insights into the functional divergence of *SQS* genes from the Cucurbitaceae family and implicate new potential roles of *SQS* positive selection, which now warrants further investigation.

## 5. Conclusions

*SQS* sequences from 10 species of Cucurbitaceae plants were cloned from which bioinformatics and phylogenetic analysis clearly suggested that Cucurbitaceae *SQS* shares a high sequence similarity. Our results indicate that (1) *SQS* sequences in plants originated very early, tracing back to bryophyte divergence to Pteridophyte and then evolved to Gymnospermae and Monocotyledonae to Dicotyledoneae; (2) 45 positively selected sites can be detected in the site model (M8) and 13 positively selected sites in the branch-site model. 196S was detected in both the site and branch-site models, 196S and 407S are present within phosphorylation sites, and 407S is located in the second transmembrane domain. 196S and 407S are considered the most significant sites for plant *SQS* and may contribute to the regulation of triterpenoid biosynthesis; and (3) positive selection and protein structure analysis were combined to explain Darwinian selection on branches and on sites in all *SQS* sequences. These changes due to positive selection were found to possess biological significance. This study will provide useful information for further research on the regulation of triterpenoid biosynthesis.

## Figures and Tables

**Table 1:** Physical and chemical property analysis in *SQS* genes in Cucurbitaceae.

Name	SS	Accession ID	Theoretical pI	Molecular weight (kD)
*Gynostemma pentaphyllum*	*GpSS*	FJ906799.1	8.33	47.90
*Momordica charantia*	*MchSS*	KX548331	8.46	47.78
*Momordica cochinchinensis*	*McSS*	KX548330	8.46	47.77
*Cucumis sativus*	*CsSS*	KX548333	7.90	47.61
*Benincasa hispida var. chieh-qua*	*BhSS*	KX548335	7.56	47.62
*Luffa acutangula*	*LaSS*	KX548336	8.46	47.60
*Cucurbita moschata*	*CmSS*	KX548332	7.18	47.59
*Trichosanthes rubriflos*	*TrSS*	KX548326	8.15	47.61
*Trichosanthes truncata*	*TtSS*	KX548327	7.90	47.59
*Trichosanthes kirilowii*	*TkSS*	KX548329	7.90	47.64

**Table 2:** Parameter estimation and likelihood ratio tests for the site model.

Model	Np	Estimate of parameters	lnL	RT pairs	df	2ΔlnL	*p*	Positively selected sites
M0: one ratio	112	*ω* = 0.12827	-29891.94					

M3: discrete	116	p0 = 0.33765, p1 = 0.39815	-28662.22	M0/M3	4	2459.44	0.001	None
		p2 = 0.26420, *ω*0 = 0.00770						
		*ω*1 = 0.09989, *ω*2 = 0.42853						

M1a: neutral	113	p0 = 0.76730, p1 = 0.23270	-29207.98	M1a/M2a	2	0.00	1.000	None
		*ω*0 = 0.08195, *ω*1 = 1.00000						

M2a: selection	115	p0 = 0.76730, p1 = 0.14030	-29207.98					
		p2 = 0.09240, *ω*0 = 0.08195						
		*ω*1 = 1.0000, *ω*2 = 1.0000						

M7: beta	113	*p* = 0.41432, *q* = 1.95163	-28621.19	M7/M8	2	11187.62	0.001	2G, 14F, 22M, 26N, 33Q, 42T, 59Q, 60P, 86E, 95I, 99R, 109S, 133E, 189S, **196S**, 200S, 250V, 265I, 277L, 354T, 357L, 369Q, 372L, 378L, 380V, 381V, 382R, 383S, 384E, 385P, 386I, 389P, 390T, 391L, 394I, 406L, **407S**, 408A, 409K, 410R, 411F, 412P, 414N, 416S, 417M (all were ∗∗)
M8: beta & *ω*	115	p0 = 1.0000, *p* = 1.09302,	-34215.80					
		*q* = 1.14565, p1 = 0.00000						
		**ω**2 = 2.53439						

lnL: loglikelihood; LRT: likelihood ratio test; *ω*2: average dN/dS ratio for sites subject to positive selection. *p* and *q*: shape parameters for the beta distribution of *ω*; p0, p1, and p2: proportions of codons subject to purifying selection, neutral evolution, and positive selection, respectively; df: degrees of freedom; 2ΔlnL: twice the loglikelihood difference of the models compared. Selection analysis by site models was performed using CodeML implemented in PAML. Bold values indicate *ω* > 1 and *p* < 0.05. Significant tests at 1% cut-off, ∗∗; posterior probability (pp) > 99%.

**Table 3:** Parameter estimation and likelihood ratio tests for the branch model.

Model	Np	Estimates of parameters	lnL	LRT pairs	df	2∆lnL	*p*	Positively selected sites (BEB)
Model 0: one ratio	112	*ω* = 0.12827	-29891.94					
		*ω*a = 156.96						
		*ω*b = 3.16						

Free model	221	*ω*c = 1.23	-29765.91	M0/free model	109	252.06	0.001	None
		*ω*d = 76.07						
		*ω*e = 670.54						
		*ω*f = 548.21						

Selection analysis by a specific free model was performed using CodeML implemented in PAML. lnL: loglikelihood; LRT: likelihood ratio test. *ω*a, *ω*b, *ω*c, *ω*d, *ω*e, and *ω*f represent the branches that dn/ds > 1.

**Table 4:** Parameter estimation and likelihood ratio tests for the two-ratio model.

Model	*P*	lnL	Parameter estimates
M0: one ratio	1	-29891.94	*ω* = 0.12827
Branch-specific models			
*Two ratios (branch a)*	2	-24451.31	*ω*0 = 0.1002, **ω** **a** = 1.1772
Two ratios (fixed *ω*a = 1)	1	-24451.32	*ω*0 = 0.1002, *ω*a = 1.0000
*Two ratios (branch b)*	2	-24444.73	*ω*0 = 0.0997, **ω** **b** = 2.9906
Two ratios (fixed *ω*b = 1)	1	-24445.24	*ω*0 = 0.0997, *ω*b = 1.0000
Two ratios (branch c)	2	-24454.01	*ω*0 = 0.1002, *ω*c = 0.4457
Two ratios (fixed *ω*c = 1)	1	-24454.03	*ω*0 = 0.1001, *ω*c = 1.0000
Two ratios (branch d)	2	-24454.53	*ω*0 = 0.1005, *ω*d = 0.0830
Two ratios (fixed *ω*d = 1)	1	-24459.38	*ω*0 = 0.1004, *ω*d = 1.0000
*Two ratios (branch e)*	2	-24450.66	*ω*0 = 0.1001, **ω** **e** = 999.0000
Two ratios (fixed *ω*e = 1)	1	-24451.12	*ω*0 = 0.1002, *ω*e = 1.0000
*Two ratios (branch f)*	2	-24453.23	*ω*0 = 0.1004, **ω** **f** = 999.0000
Two ratios (fixed *ω*f = 1)	1	-24453.49	*ω*0 = 0.1004, *ω*f = 1.0000

Selection analysis by a branch model was performed using CodeML implemented in PAML. lnL: loglikelihood; LRT: likelihood ratio test; *P*: parameters in the calculation.

**Table 5:** Parameter estimation and likelihood ratio tests for the branch-site model.

Model	Np	Estimates of parameters	lnL	LRT pairs	df	2*∆*lnL	*p*	Positively selected sites (BEB)
BSa1	114	f: *ω*0 = 0.07521, *ω*1 = 1.00000, *ω*2a = 1.00000, *ω*2b = 1.00000						None
		p0 = 0.16610, p1 = 0.02926, p2a = 0.68412, p2b = 0.12052						

BSa0_fix	115	f: *ω*0 = 0.07521, *ω*1 = 1.00000, *ω*2a = 2.53464, *ω*2b = 2.53464	-24079.01	BSa1/BSa0-fix	1	4.18	**0.0409**	
		p0 = 0.02224, p1 = 0.00386, p2a = 0.82997, p2b = 0.14393	-24076.92					13D pp = 0.950^∗^, **196S** **p** **p** = 0.959, 278K pp = 0.950^∗^

BSb1	114	f: *ω*0 = 0.07495, *ω*1 = 1.00000, *ω*2a = 1.00000, *ω*2b = 1.00000						
		p0 = 0.00000, p1 = 0.00000, p2a = 0.85217, p2b = 0.14782	-24074.35	BSb1/BSb0-fix	1	7.02	**0.00806**	None

BSb0_fix	115	f: *ω*0 = 0.07493, *ω*1 = 1.00000, *ω*2a = 4.18844, *ω*2b = 4.18844	-24070.84					52A, 157K, 171Y, 180L, 203L, 226M, 289P, 333G pp = 0.981^∗^
		p0 = 0.40765, p1 = 0.07083, p2a = 0.44432, p2b = 0.07720						

BSe1	114	f: *ω*0 = 0.07520, *ω*1 = 1.00000, *ω*2a = 1.00000, *ω*2b = 1.00000		BSe1/BSe0-fix	1	6.64	**0.00997**	None
		p0 = 0.00007, p1 = 0.00001, p2a = 0.85016, p2b = 0.14976	-24079.9					

BSe0_fix	115	f: *ω*0 = 0.07520, *ω*1 = 1.00000, *ω*2a = 999.00000, *ω*2b = 999.00000	-24076.58					37W pp = 0.966^∗^, 308R pp = 0.960^∗^
		p0 = 0.00116, p1 = 0.00020, p2a = 0.84911, p2b = 0.14952						

BSf1	114	f: *ω*0 = 0.07526, *ω*1 = 1.00000, *ω*2a = 1.00000, *ω*2b = 8.15518						
		p0 = 0.00000, p1 = 0.00000, p2a = 0.85030, p2b = 0.14970	-24079.91	BSf1/BSf0-fix	1	3.30	0.06928	None

BSf0_fix	115	f: *ω*0 = 0.07541, *ω*1 = 1.00000, *ω*2a = 632.33181, *ω*2b = 632.33181	-24078.26					204F pp = 0.847
		p0 = 0.00000, p1 = 0.00000, p2a = 0.85033, p2b = 0.14967						

Selection analysis by a branch-site model was performed using CodeML implemented in PAML. BS: branch site. The significant tests at 5% cut-off are labeled with ∗. Posterior probability (pp) > 95%. Bold: *p* < 0.05. BS0-fix: fixed *ω* = 0; BS1: fixed *ω* = 1.

## Data Availability

Nine SS sequences of Cucurbitaceae plants, cloned by our laboratory, have uploaded on GenBank (http://www.ncbi.nlm.nih.gov/) and these sequences will released with the publication of the article. The rest SS sequences of cDNA and amino acid were downloaded from the GenBank and the UniProt (http://www.uniprot.org/). All the accession numbers of sequences involved in article were list in the additional files.

## Abbreviations

*SQS*: Squalene synthase
LB: Luria-Bertani medium
*GpSS*: Gynostemma pentaphyllum squalene synthase
*PnSS*: Panax notoginseng squalene synthase
*GARD*: Genetic Algorithm for Recombination Detection
*PP*: Posterior probability
*RACE*: Rapid amplification of cDNA ends
*BEB*: Bayes empirical Bayes
*LRT*: Likelihood ratio test
*dN*: Nonsynonymous
*dS*: Synonymous.

## Supplementary Materials

Supplementary Materials The datasets of squalene synthase in plants used in the research, including the accession number in GenBank, gene resource, family, cDNA bp, ORF length, and amino acid length.
